# Scalable and Efficient Deep Learning-Based Pipeline for Mitotic Detection and Analysis in Pathology Images

**DOI:** 10.3390/cancers18111807

**Published:** 2026-06-01

**Authors:** Xuan Qi, Dominic LaBella, Thomas Sanford, Ismail Turkbey, Maxwell Lee

**Affiliations:** 1 Laboratory of Cancer Biology and Genetics, Center for Cancer Research, National Cancer Institute, National Institutes of Health, Bethesda, MD 20892, USA; 2 Department of Radiation Oncology, Duke University Medical Center, Durham, NC 27710, USA; dominic.labella@duke.edu; 3 University of Hawaii Cancer Center, University of Hawaii at Manoa, Honolulu, HI 96813, USA; 4 Molecular Imaging Branch, Center for Cancer Research, National Cancer Institute, National Institutes of Health, Bethesda, MD 20892, USA; ismail.turkbey@nih.gov

**Keywords:** mitosis, atypical mitosis, detection, classification, pathology, deep learning, survival analysis, efficiency

## Abstract

**Background:** Accurate and efficient analysis of mitotic figures in whole-slide images (WSIs) is essential for tumor grading and prognosis. **Methods:** In this work, we present a three-stage pipeline for WSI-scale mitosis analysis that balances accuracy with clinical throughput: (1) a YOLOv11-based detector to propose mitosis candidates; (2) an ultra-lightweight classifier to refine detections and suppress false positives; and (3) a downstream classifier to distinguish atypical from normal mitoses for deeper biological insight. **Results:** In benchmark datasets, the two-stage detector improves F1 over detection-only baselines, while the atypical/normal module achieves strong accuracy, demonstrating cross-domain generalization. We further perform a proof-of-concept survival analysis on early-stage (I–II) cases from the TCGA-BRCA cohort, suggesting that mitosis-derived features may provide modest incremental prognostic information beyond the clinical baseline and nuclei features. **Conclusions:** Overall, the method delivers accurate detection, robust atypical mitosis classification, and high efficiency, processing gigapixel WSIs in minutes on a single GPU, positioning it for large-scale translational studies and future clinical workflow validation.

## Simple Summary

Mitotic figures are important markers of tumor growth, but counting them manually in whole-slide pathology images is time-consuming and subject to observer variability. We developed an efficient artificial intelligence pipeline that detects mitotic figures, suppresses likely false-positive candidates, and further classifies mitotic figures as atypical or normal. The method was evaluated on public mitosis detection and atypical mitosis classification benchmarks and applied to breast cancer whole-slide images from the TCGA-BRCA cohort. Our results show that the pipeline achieves strong detection and classification performance while processing gigapixel slides within minutes on a single GPU. In an exploratory survival analysis of early-stage TCGA-BRCA cases, mitosis-derived features showed modest additional prognostic information beyond clinical and nuclei morphology features. These findings suggest that efficient automated mitosis analysis may support large-scale pathology studies and motivate further validation in independent clinical cohorts.

## 1. Introduction

Mitosis is a key stage of the cell cycle that represents active cell division and serves as a direct measure of tumor proliferative activity. In histopathology, the mitotic count is a crucial component of tumor grading systems, such as the most widely used Nottingham Grading System [1] for breast cancer. High mitotic activity often indicates aggressive tumor behavior and is associated with poorer clinical outcomes [2,3].

Traditionally, pathologists perform mitotic analysis manually by selecting regions of interest, typically high-power fields, and counting the number of mitotic figures within each field [1,4]. However, this manual approach has several major limitations. First, modern digitally scanned whole-slide images (WSIs) are gigapixel in scale, especially under high magnification like 40×, making exhaustive visual inspection extremely time-consuming. In addition to the size issue, tumor heterogeneity makes the manual selection of high-power fields subjective and potentially biased [5,6]. Furthermore, domain shifts [7] such as staining variations, scanner differences, and the morphological complexity of mitotic figures make manual mitotic analysis inconsistent.

Hence, in this work, we propose a deep learning-based pipeline for mitosis detection and classification to address the above challenges. Our pipeline consists of three stages: (1) mitosis candidate detection, (2) mitosis versus non-mitosis classification, and (3) atypical mitotic figure (AMF) versus normal mitotic figure (NMF) classification. We train the Stage 1 and Stage 2 models with the MIDOG++ dataset [8] and the Stage 3 model with the MIDOG 2025 Challenge Track 2 (Atypical Mitosis Classification) dataset [9]. We validate detection performance on the MIDOG++ test split and report AMF classification results on the official test set of the MIDOG 2025 Challenge Track 2. Furthermore, we evaluate our pipeline on the TCGA-BRCA [10] cohort to assess the translational and prognostic relevance of mitotic activity.

The contributions of this work are summarized as follows:

- **Scalability and Efficiency.** We present a deep learning-based pipeline for mitosis detection and classification, enabling reliable analysis of large-scale clinical datasets.
- **Generalization and Clinical Relevance.** We demonstrate the robust generalization of our pipeline across multiple tumor types and evaluate the clinical relevance of mitotic activity. By integrating a downstream survival analysis on the TCGA-BRCA cohort, this work explores the feasibility of mitosis density as a candidate prognostic feature in early-stage TCGA-BRCA.
- **Accessible AI, Real-World Applicability.** Rather than trading marginal performance gains for excessive computational cost, our pipeline is suitable for large-scale research use and future clinical workflow evaluation, with the potential to support more efficient pathology workflows after appropriate validation.

## 2. Related Works

### 2.1. Mitosis Detection

Deep learning-based mitosis detection can be broadly categorized into three major types: patch-level classification, one-stage detection, and two-stage detection.

Early works framed mitosis detection as binary, patch-based classification (mitosis vs. non-mitosis) [11,12]. However, patch-level labels are a poor proxy for instance counting, especially when a positive tile contains multiple mitotic figures. Moreover, localization is inherently imprecise: predictions depend on tile size, stride, and border placement, which can cause both misses and double counting.

Later, object detection reframes the task as predicting bounding boxes around mitotic figures. For detection-based methods, RetinaNet [13] with focal loss addresses extreme class imbalance and was adopted as the official reference model in the MIDOG 2022 challenge [14]. On top of this, Wang et al. [15] augmented RetinaNet with a foreground cue and a tumor-region classifier, improving cross-domain generalization. Ardac et al. [16] further modified the detection transformer (DETR) [17] with improved feature representation. Other than detection, segmentation models have also been applied to mitotic figure detection. For example, Li et al. [18] adopted a weakly supervised approach: using only centroid (point) annotations instead of instance-level masks, they converted each into a soft segmentation target via concentric circles and trained an FCN with a custom loss.

Moreover, adding a second-stage classifier has become a popular design. Fick et al. [19] employed Mask-RCNN [20] to segment mitotic figures and applied an ensembled second-stage classifier (ResNet-50 [21] and DenseNet-201 [22]). Similarly, Jahanifar et al. [23] proposed another design that first generates high-recall candidates with a reduced-resolution segmentation model, followed by a classifier working at 40× resolution that prunes mimickers. This design achieves strong cross-domain performance but carries a structural limitation: small or faint mitoses may be missed at the low-resolution candidate stage and are therefore missed downstream. Recently, foundation models have also been applied as the first stage. Shen et al. [24] used Segment Anything Model (SAM) [25] to segment all nuclei/cell-scale objects in WSI. Then, a fine-tuned ResNet-18-based classifier is applied to distinguish mitotic figures. This approach achieves strong performance, but the computational cost is expensive: even a base-scale SAM model (SAM-B) requires 487 GFLOPs for a single forward pass of a 1024 × 1024 tile, solely for its encoder part. Hence, we believe that a good balance between performance and efficiency is critical for broader clinical adoption and accessibility.

### 2.2. Atypical Mitosis Classification

Standard morphologic criteria for distinguishing atypical mitotic figures (AMFs) from normal mitoses and from impostors are summarized by Donovan et al. [26]. Recent clinical studies further report that a higher AMF count is associated with more aggressive tumor behavior and poorer outcomes [27,28]. Therefore, beyond mitosis detection, classifying AMFs versus normal mitotic figures (NMFs) represents a deeper and clinically meaningful level of analysis. However, this is a new and challenging topic: AMFs are relatively rare compared to NMFs, causing severe class imbalance during model training, and their morphological differences are subtle and hard to distinguish.

To bridge this gap, MIDOG 2025 [9] formalizes AMF classification as the Track 2 task: binary classification of cropped mitotic patches drawn from multiple centers and tumor types, with balanced accuracy (BA) (4) as the primary metric to handle class imbalance. The track emphasizes cross-domain generalization and robustness to subtle morphological differences. We submitted our method to this track and incorporated the resulting model as the Stage 3 AMF/NMF classifier in our pipeline.

## 3. Methods

### 3.1. Pipeline Overview

**Figure 1:**
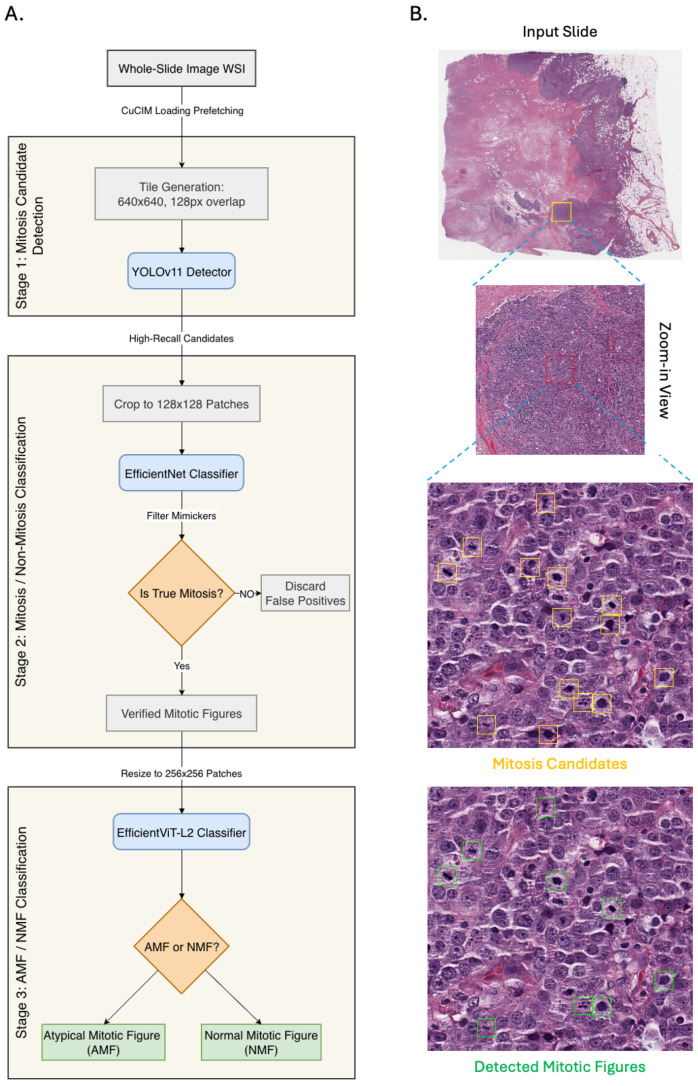
Overview of the mitosis analysis pipeline. (**A**) Proposed three-stage framework. Whole-slide images are loaded with cuCIM and tiled into overlapping 640×640 pixel regions. A YOLOv11 detector identifies mitosis candidates, which are then filtered by an EfficientNet classifier to suppress false-positive candidates. In Stage 3, an EfficientViT-L2 model is applied as a classifier for atypical mitotic figures (AMFs) or normal mitotic figures (NMFs). (**B**) Sample visualization of the mitosis detection. Yellow boxes indicate mitosis candidates generated in Stage 1, and green boxes indicate verified mitotic figures retained after Stage 2.

Our mitosis analysis pipeline, illustrated in Figure 1, consists of three stages: (1) mitosis candidate detection, (2) mitosis verification, and (3) atypical versus normal mitotic figure classification. In Stage 1, whole-slide images are loaded using the cuCIM [29] GPU-accelerated library and divided into overlapping tiles of 640×640 pixels with a 128-pixel overlap. A YOLOv11-based detector then scans these tiles to identify high-recall mitosis candidates. In Stage 2, each candidate region is cropped into a 128×128 patch and evaluated by an EfficientNet [30] classifier, which distinguishes true mitotic figures from non-mitotic mimickers and suppresses false positives from the initial detection step. Verified mitotic figures are subsequently resized to 256×256 patches and passed to an EfficientViT-L2-based classifier in Stage 3, where they are further categorized as atypical mitotic figures (AMFs) or normal mitotic figures (NMFs), enabling fine-grained morphologic characterization. Figure 1A summarizes the computational workflow, while Figure 1B shows an example visualization of candidate detection and final mitotic figure identification.

### 3.2. Models

#### 3.2.1. Mitosis Detection Models

We compared several representative and widely used efficient object detection models, including Faster R-CNN [31] with a ResNet-50 FPN backbone [32] and RT-DETR [33], shown in Table 1. Among these, YOLOv11 emerged as the most lightweight, requiring ∼3–9× fewer GFLOPs than RT-DETR and Faster R-CNN, while maintaining comparable accuracy. This efficiency enables practical whole-slide processing for large-scale research use and future clinical workflow evaluation. Hence, we adopt YOLOv11 pretrained on the COCO dataset [34] and fine-tune it on mitosis data. To further suppress false positives, we incorporate a second-stage EfficientNet-based classifier [30], which refines detections by distinguishing true mitotic figures from visually similar non-mitotic structures.

#### 3.2.2. AMF Classification Model

Vision Transformers (ViTs) are highly effective for image classification, as self-attention captures both local features and long-range context. For mitosis classification, however, efficiency is critical given the scale of whole-slide images (WSIs). To balance accuracy and efficiency, we adopt EfficientViT [35], a hybrid architecture that integrates convolutional and Transformer modules and employs cascaded linear attention to reduce computation and memory. Specifically, we use an ImageNet-pretrained EfficientViT-L2 as the AMF/NMF classifier with 256×256 inputs, and fine-tune it on our AMF/NMF dataset. Our choice is twofold: (1) EfficientViT-L2 attains 85.37% ImageNet top-1 accuracy with 64 M parameters and only 18.2 GFLOPs, offering an excellent performance-efficiency trade-off; and (2) this larger-scale variant provides greater modeling capacity to capture the subtle morphological differences between AMF and NMF discussed in Section 2. To further improve generalization to unseen domains, we employ a five-fold ensemble strategy for training the AMF/NMF classifier. Specifically, we partition each cancer type into five folds, creating five subsets of the whole dataset. We train five independent models on these splits, and the final prediction is obtained by averaging the outputs across all five models.

### 3.3. Training

#### 3.3.1. Data Sampling

**Figure 2:**
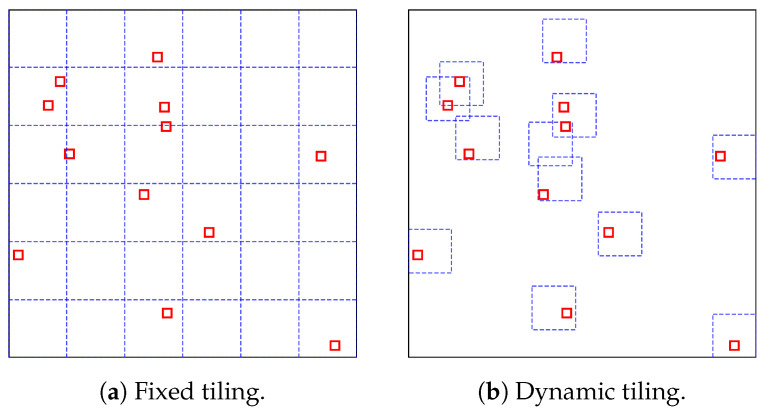
Dynamic training tiles sampling strategy. The ground-truth bounding boxes of mitotic figures are in red, and the sampled tiles are in blue.

The conventional fixed tiling is suitable for evenly distributed targets such as nuclei or cells; however, mitotic figures are sparse and highly random in spatial distribution. Hence, a *dynamic tiling strategy* that regenerates cropped samples at each training epoch is applied in this work. As illustrated in Figure 2a, fixed tiling often produces excessive negative tiles without mitotic figures and may also truncate mitoses along tile borders, leading to class imbalance. To address these issues, we adopt a dynamic sampling strategy, which is shown in Figure 2b. For positive samples, tiles are cropped around the ground-truth bounding boxes of mitotic figures, allowing one or multiple targets to appear within a single tile. For negative samples, tiles are cropped from regions that exclude annotated mitoses. This sampling is performed dynamically at every training epoch, ensuring that the same mitotic figure can appear at different locations within a sample tile, thereby improving generalization and reducing overfitting. The implementation of this dynamic sampling approach follows the reference code provided in the MIDOG 2025 Guide [36].

#### 3.3.2. Data Augmentation

To improve generalization, especially to unseen domains and staining conditions, we applied a stain-based deconvolution augmentation method [37] as the main color augmentation strategy for all three stages of the pipeline. Specifically, H&E stain jittering was used to perturb images in the Hematoxylin and Eosin color space, rather than treating pathology images simply as generic RGB images. This content-aware augmentation more faithfully simulates staining variability caused by differences in tissue preparation, scanner acquisition, and institutional protocols, thereby improving robustness to domain shift.

In addition to H&E stain augmentation, we applied spatial and image-quality augmentations during training. These included horizontal flipping, vertical flipping, random 90-degree rotation, defocus blur, and Gaussian blur. For the detection model, the corresponding bounding-box coordinates were transformed consistently with the image augmentations. Together, these augmentations were designed to improve robustness to orientation variation, local morphology variation, and mild microscope or scanning artifacts while preserving the biological appearance of mitotic figures.

#### 3.3.3. Other Details

Training is conducted using the PyTorch framework (version 2.8.0). The main software environment included Ultralytics 8.3.228, torchvision 0.23.0, timm 1.0.22, cuCIM-cu12 25.10.0, OpenSlide-python 1.3.1, Albumentations 2.0.8, NumPy 2.2.6, Matplotlib 3.10.6, SciPy 1.15.3, and lifelines 0.30.0. For training the first-stage model, we fine-tuned YOLOv11 detectors (n/s/m/l) from pretrained weights using Ultralytics 8.3.228 and mixed-precision (fp16) training with AdamW (initial learning rate $1\times 10^{-3}$, weight decay $5\times 10^{-4}$) and a cosine annealing schedule. We monitored validation performance using an exponential moving average (EMA) model and selected the best checkpoint according to mAP@0.5, with early stopping (patience of 15 epochs) applied after epoch 60.

For the second-stage mitosis/non-mitosis classifier, we trained the EfficientNet models on the same slide-level split used for Stage 1, using 128×128 candidate-centered patches that included both annotated mitotic figures and hard-negative non-mitotic mimickers. We additionally evaluated true 256×256 candidate-centered crops for the Stage 2 EfficientNet-B0 classifier, cropped from the original high-resolution image around each Stage 1 candidate location rather than generated by resizing 128 × 128 patches. This setting achieved 85.23% accuracy and an F1-score of 0.8512, compared with 84.69% accuracy and an F1-score of 0.85 for the 128×128 setting. However, increasing the crop size from 128 × 128 to 256 × 256 increases the input area by fourfold, increasing the approximate EfficientNet-B0 computation from 0.25 to about 1.0 GFLOPs per candidate. Because Stage 1 is intentionally operated as a high-recall detector and generates many candidate regions per WSI, this additional candidate-level overhead is substantial at the whole-slide scale. Moreover, larger candidate-centered crops may include nearby mitotic figures, non-mitotic mimickers, or clustered nuclei, making candidate-level mitosis/non-mitosis verification less focused. Therefore, we retained 128 × 128 crops for Stage 2 as the preferred accuracy-efficiency trade-off. The classifier was implemented in PyTorch 2.8.0 with timm 1.0.22 and used the same augmentation pipeline described above, so that the model learned to reject common false-positive patterns while remaining robust to cross-domain appearance variation.

For training the AMF/NMF classifier, because the AMF class is substantially underrepresented relative to the NMF class, we use focal loss [13] and a weighted sampling strategy.

During inference, the input WSI is divided into 640×640 tiles with a 128-pixel overlap. Non-Maximum Suppression (NMS) is then applied to the raw model outputs to remove overlapping bounding boxes and eliminate duplicate detections of the same mitotic figure occurring in tile overlap regions. For the WSI loading and preprocessing, we use the Nvidia cuCIM [29] library (cuCIM-cu12 version 25.10.0), which is highly optimized compared to the OpenSlide library [38]. In addition, sliding-window data prefetching is performed in parallel with model inference, minimizing GPU idle time and achieving significant speedup.

### 3.4. Survival Analysis on TCGA-BRCA Cohort

To further explore the clinical relevance of our pipeline, we applied it to the TCGA-BRCA dataset [10], the largest publicly available breast cancer cohort containing approximately 1000 cases.

First, mitosis density was evaluated for prognostic relevance in early-stage (I–II) breast cancer patients, using disease-specific survival (DSS) as the clinical endpoint. We analyzed 3-year and 4-year survival outcomes, dividing patients into high and low-density groups based on the median mitosis density. Patients without a DSS event by 36 or 48 months were administratively censored. Kaplan–Meier (KM) curves [39] were used to visualize survival probabilities and assess group separation. The KM and Cox analyses used slightly different sample counts because the fixed-horizon analyses required complete information for different combinations of DSS follow-up, baseline covariates, and extracted pathology features at each time horizon.

Additionally, we used Cox proportional hazards models to further investigate the prognostic significance of mitotic density. We first defined a clinical baseline model that included age, stage, and cancer subtype. We then assessed whether adding pathological features provided incremental prognostic value beyond this baseline. Specifically, Model 2 added nuclei morphology features, defined as the mean and standard deviation of the sizes of the top 10% largest tumor nuclei, reflecting pathology practice that emphasizes enlarged and abnormal nuclei [1,40]. Model 3 further added mitosis density, defined as the number of detected mitotic figures divided by tumor area. This nested design allowed us to quantify whether nuclear morphology and mitotic activity contributed complementary prognostic information beyond standard clinical variables.

To quantify the tumor area, we first applied the Hover-Next nuclei segmentation tool [41], which simultaneously yields nuclei type classification results. The whole-slide image is then divided into small tiles of size 64×64 pixels, and the number of nuclei within each tile is counted. A tile is labeled as part of the tumor region if it contains at least one tumor nucleus. The tumor area mask is subsequently constructed from all tumor tiles, and the mitosis density is defined as the number of mitotic figures within the tumor area divided by the total tumor area.

## 4. Experiments and Results

### 4.1. Experimental Settings

#### 4.1.1. Training Dataset

Following the official dataset split configuration, 111 slides are reserved for testing and 392 slides for training. The official train/test partition was obtained from the MIDOG++ GitHub repository using the provided datasets_xvalidation.csv split file [42]. From the official training split, 90% (350 slides) are used for model training and 10% (42 slides) for validation. All training, validation, and test partitions were therefore defined at the slide/case level. For Stage 1 and Stage 2, all sampled tiles, candidate-centered crops, and hard-negative samples inherited the split assignment of their source slide; no samples derived from held-out test slides were used for training, validation, model selection, or threshold tuning. The tumor-type and slide distribution for the combined dataset is summarized in Table 2.

For the second-stage mitosis/non-mitosis classifier, we trained the EfficientNet models on the same slide-level split used for Stage 1, using 128 × 128 candidate-centered patches that included both annotated mitotic figures and hard-negative non-mitotic mimickers. Recent cell-centric pathology work has shown that larger patches can provide additional context for some tasks; for example, Devnath et al. reported that 256 × 256 nucleus-centered patches outperformed 128 × 128 patches for recognizing epithelial cells in prostatic glands [43]. Motivated by this patch-size consideration, we additionally evaluated true 256 × 256 candidate-centered crops for the Stage 2 EfficientNet-B0 classifier, cropped from the original high-resolution image around each Stage 1 candidate location rather than generated by resizing 128 × 128 patches.

For the third-stage Atypical Mitotic Figure (AMF) versus Normal Mitotic Figure (NMF) classification, we utilize the MIDOG 2025 Challenge Track 2 dataset [9], which includes the MIDOG 2025 Atypical Training Set [44] and the AMi-Br dataset [45]. For Stage 3, duplicate samples were removed before dataset combination, and the official MIDOG 2025 final test set was used only for final evaluation; it was not used for training, validation, model selection, threshold tuning, or ensemble construction, as summarized in Table 3.

#### 4.1.2. Metrics

To comprehensively evaluate our models, we report both the F1-score and balanced accuracy (BA) as primary metrics. Precision and recall are defined in (1), where TP denotes true positives, FP false positives, FN false negatives, and TN true negatives. The F1-score, which is the harmonic mean of precision and recall, is calculated as (2).

(1) $$\begin{array}{c} \mathrm{Precision}=\frac{TP}{TP+FP};\;\mathrm{Recall}=\frac{TP}{TP+FN} \end{array}$$

(2) $$F1=2\times \frac{\mathrm{Precision}\times \mathrm{Recall}}{\mathrm{Precision}+\mathrm{Recall}}$$

Balanced accuracy (BA) is particularly useful for imbalanced data, like the AMF/NMF classification. It is the average of sensitivity (recall) and specificity, where specificity is defined as:

(3) $$\mathrm{Specificity}=\frac{TN}{TN+FP}$$

(4) $$\mathrm{BA}=\frac{\mathrm{Recall}+\mathrm{Specificity}}{2}$$

### 4.2. Experimental Results

#### 4.2.1. Mitosis Detection Results

We first compare four YOLOv11 variants as the first-stage detector on the MIDOG++ test set. For the second stage, we use EfficientNet-B0 as a mitosis/non-mitosis classifier, which adds only 0.25 GFLOPs of computational overhead. Evaluation uses an IoU threshold of 0.5, with confidence thresholds of 0.1 for Stage 1 and 0.5 for Stage 2. As shown in Table 4, all variants achieve high recall at the detection stage, demonstrating strong sensitivity for capturing mitotic figures and minimizing missed true positives. High recall is important for mitosis candidate generation because missed mitoses can lead to underestimation of proliferative activity. To mention here, because Stage 3 performs AMF/NMF subclassification only after verified mitotic figures have been detected, it is evaluated separately in Section 4.2.3 and is not included as an additional mitosis-detection filter in this ablation.

To further analyze the second stage, Table 5 compares EfficientNet against the widely used ResNet. Notably, we observe that models that are ∼2–4× lighter than ResNet-18 can achieve comparable or even higher mitosis/non-mitosis classification performance.

The second-stage classifier delivers substantial performance gains: precision increases dramatically (e.g., 0.41 to 0.71 for YOLOv11-l), and the overall F1-score improves consistently across all variants while maintaining high recall (∼0.78–0.82). This precision-recall trade-off effectively suppresses likely false positives propagated from the first stage without sacrificing sensitivity. Importantly, these improvements come without significant computational overhead: even the small and nano variants maintain competitive accuracy, making the pipeline practical for large-scale research use and future clinical workflow evaluation. In summary, the two-stage framework achieves both high accuracy and computational efficiency, demonstrating that strong detection performance is attainable with minimal resource requirements.

**Figure 3:**
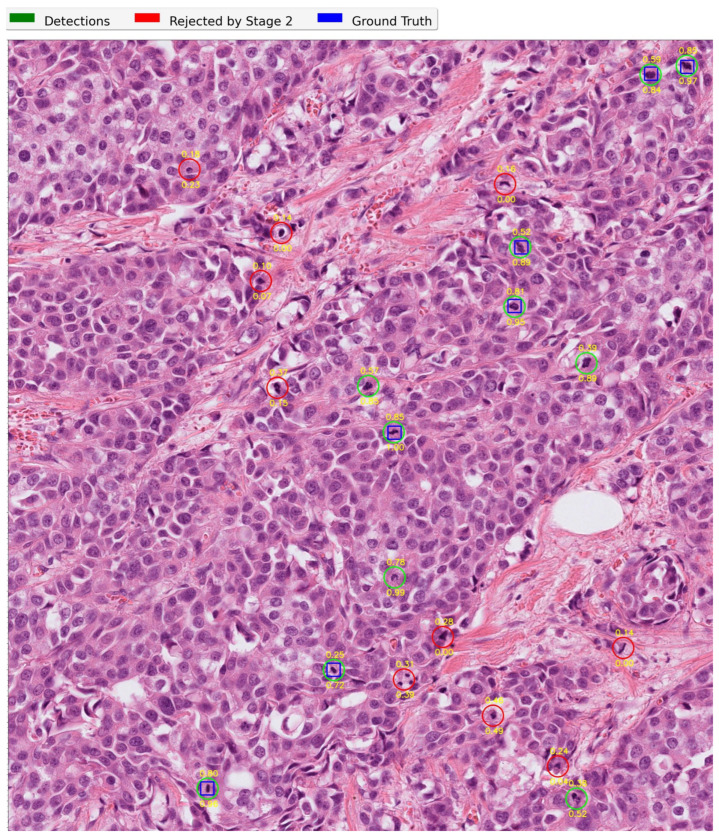
Example from a MIDOG++ test slide showing how Stages 1 and 2 detect true mitotic figures while suppressing likely false-positive candidates. Ground-truth mitoses are boxed in blue, circles mark Stage 1 candidate detections, and Stage 1 confidence scores are shown above them. Candidates with high Stage 2 confidence are retained as true mitotic figures and marked in green; otherwise, they are suppressed from the automated count and marked in red. Thresholds: 0.1 for Stage 1 and 0.5 for Stage 2.

Figure 3 shows a representative example from a MIDOG++ test slide, illustrating how the Stage 2 mitosis classifier acts as a computational filtering step to suppress false positives from Stage 1. Quantitatively, filtering these unmatched candidates improves the overall F1-score. Qualitatively, the false-positive candidates mainly follow two patterns that can confuse the detector: (1) isolated nuclei with mitosis-like appearance, and (2) clusters of closely adjacent nuclei that visually resemble a single mitotic figure.

**Figure 4:**
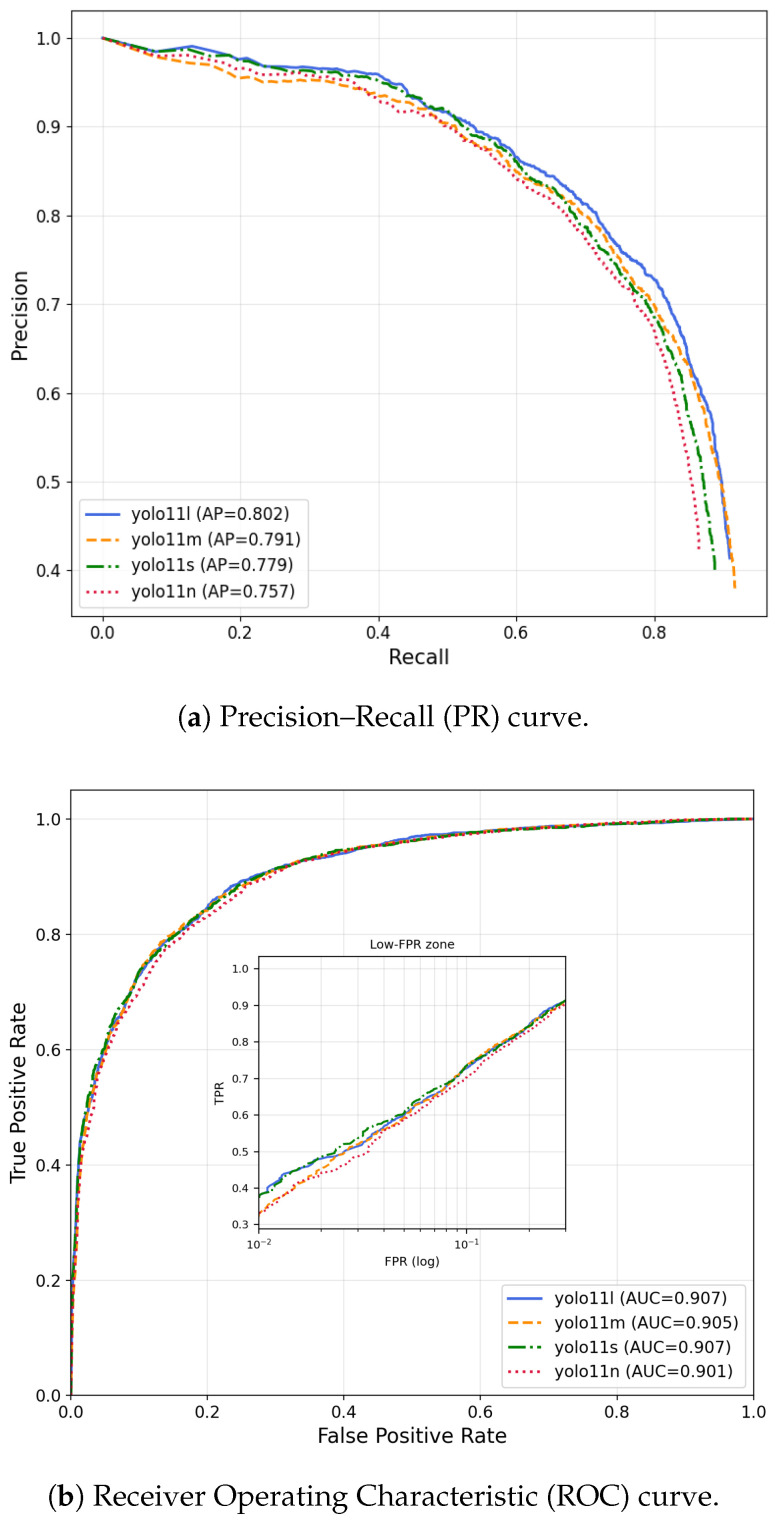
Precision–Recall and ROC curves on the MIDOG++ test set with different YOLOv11 variants as the first-stage detector and EfficientNet-B0 as the second-stage classifier. (**a**) Precision–Recall curves, with average precision (AP) reported for each model variant. (**b**) Receiver Operating Characteristic (ROC) curves, with ROC-AUC reported for each model variant. The main ROC panel uses the conventional linear false-positive-rate (FPR) axis from 0 to 1. The inset highlights the low-FPR operating region using a logarithmic FPR axis to better visualize model behavior at low false-positive rates.

Figure 4 shows that the two-stage pipeline performs consistently across YOLOv11 variants on the MIDOG++ test set. Figure 4a shows the Precision–Recall curves, where YOLOv11-l achieves the best AP (0.802), followed by YOLOv11-m, YOLOv11-s, and YOLOv11-n, indicating the strongest Precision–Recall trade-off among the four variants. Figure 4b shows the ROC curves, where all variants demonstrate similar candidate-level discrimination, with ROC-AUC values above 0.90 and YOLOv11-l again performing best (0.907). Overall, these results support Table 4: YOLOv11-l gives the highest accuracy, while smaller YOLOv11 models remain competitive with lower computational cost.

#### 4.2.2. Performance Comparison with Other Mitosis Detection Approaches

To further contextualize detection performance and computational practicality, we compared the proposed pipeline with representative published approaches evaluated on the MIDOG++ benchmark: the official RetinaNet baseline from that dataset paper [8], a recent Faster R-CNN-based approach [46], and OMG-Net, a recent SAM-based foundation-model approach [24]. It is important to note that RetinaNet, Faster R-CNN, and our pipeline are all trained exclusively on the MIDOG++ training split, whereas OMG-Net was trained on a larger multi-source dataset and leverages a SAM-based foundation model backbone with additional mask curation. As shown in Table 6, within the group of MIDOG++-only methods, the proposed YOLOv11-L + EfficientNet-B0 pipeline achieved a pooled test-set F1 of 0.76 (Table 4) and a mean domain-wise F1 of 0.73 on the official MIDOG++ test split, matching the Faster R-CNN mean domain-wise F1 and slightly exceeding the RetinaNet baseline.

Notably, despite being trained solely on MIDOG++ and using a lightweight detector-plus-classifier design, our pipeline achieves a mean domain-wise F1 of 0.73, only 0.04 points below OMG-Net (0.77). Given that OMG-Net benefits from a larger multi-source training set and a computationally intensive SAM ViT-H foundation model backbone, this narrow gap highlights the competitive accuracy of our approach under a substantially more constrained training and inference budget. In two of the three canine tumor types shared with OMG-Net’s reported domains, our pipeline achieves tied-best F1, further supporting its effectiveness relative to a cutting-edge foundation-model-based method.

The main advantage of the proposed pipeline is its accuracy–efficiency trade-off. Rather than treating mitosis detection as a pure benchmark-optimization problem, our goal is to develop a practical WSI-scale system that provides sufficient accuracy while remaining computationally accessible. As shown in Table 7, the YOLOv11-L detector requires substantially less computation than the RetinaNet R50-FPN, Faster R-CNN R50-FPN, and the SAM ViT-H proposal backbone used by OMG-Net. Compared with RetinaNet and Faster R-CNN, YOLOv11-L reduces detector-level computational cost by approximately 43% and 68%, respectively, while maintaining comparable mean domain-wise F1. Compared with the area-normalized SAM ViT-H encoder estimate, YOLOv11-L requires approximately 13.4× lower first-stage computation. Furthermore, smaller YOLOv11 variants provide an additional efficiency range, requiring approximately 1.3× to 13.4× less detector-level computation than YOLOv11-L. This scalability allows the pipeline to be adapted to different deployment constraints, from higher-accuracy server-side inference to lightweight, high-throughput WSI screening. Importantly, this design philosophy supports more accessible AI for pathology: models that are accurate enough to be clinically useful, but efficient enough to be deployed broadly across research and healthcare settings with limited computational resources.

These results support the design goal of developing a mitosis detection pipeline that is not only accurate, but also fast and computationally efficient enough for practical WSI-scale deployment.

#### 4.2.3. AMF/NMF Classification Results

For our AMF/NMF classifier, we submitted results to the MIDOG 2025 Challenge [9] (Track 2: Atypical Mitosis Classification). The model was trained using the combined dataset described in Table 3. We employed a 5-fold cross-validation strategy to train five independent models and used an ensemble averaging approach to obtain the final predictions. On the comprehensive final test set consisting of 3800 images from 12 different cancer types, our method achieved an overall ROC-AUC of 0.962 and a balanced accuracy of 0.897, as shown in Table 8. These strong results confirm the robustness and generalization ability of our approach across multiple cancer types and highlight its potential for reliable, clinically applicable mitosis classification.

#### 4.2.4. Survival Analysis

**Figure 5:**
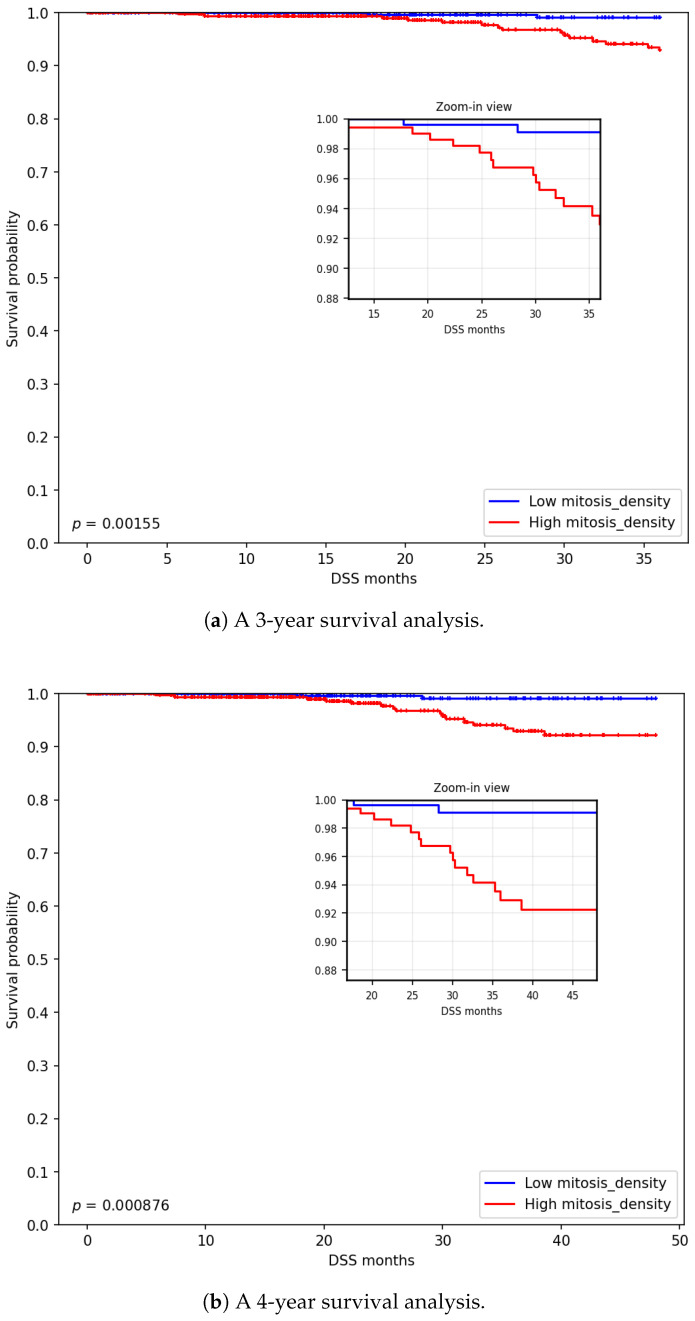
Survival analysis performed on the TCGA-BRCA early-stage (I and II) samples, stratified by mitosis density processed with our pipeline. (**a**) A 3-year and (**b**) 4-year disease-specific survival (DSS) curves, with groups split by median mitosis density. Patients with high mitosis density (red) show lower DSS compared to low mitosis density (blue).

Early-stage breast cancer is a clinically important setting, as more accurate risk classification and prediction at diagnosis may yield the greatest benefit for patient survival, follow-up planning, and treatment decision-making. Hence, we first performed a Kaplan–Meier (KM) survival analysis on early-stage (I–II) cases from the TCGA-BRCA cohort (*n* = 755), stratified by mitosis density estimated using our pipeline. Patients were divided into high-density and low-density groups based on the median mitosis density. As shown in Figure 5a, the 3-year KM survival curve showed a clear separation between the two groups, with a significant difference in disease-specific survival (*p* = 0.00155). The survival gap persisted in the 4-year analysis (*p* = 0.000876), as shown in Figure 5b, indicating a consistent mid-term prognostic effect. These exploratory Kaplan–Meier results suggest that higher mitotic activity is associated with poorer disease-specific survival in early-stage TCGA-BRCA, consistent with the clinical expectation that tumors with higher mitotic activity tend to exhibit more aggressive behavior.

For Cox model analysis, the results in Table 9 show a consistent pattern in which computational pathology features provide modest incremental prognostic discrimination beyond the clinical baseline. At 36 months, the clinical baseline model achieved a C-index of 0.769, which increased to 0.781 after adding nuclei morphology features and further to 0.802 after incorporating mitosis density. At 48 months, a similar trend was observed, with the C-index improving from 0.777 for the clinical baseline to 0.787 after adding nuclei morphology features and further to 0.802 after incorporating mitosis density. However, the bootstrap confidence intervals were relatively wide and overlapping across models, consistent with the limited number of disease-specific events in this fixed-horizon analysis.

Taken together, these exploratory Cox model results suggest that mitosis density may provide modest incremental prognostic information beyond clinical variables and nuclei morphology in early-stage TCGA-BRCA. However, given the limited number of disease-specific events and the proof-of-concept nature of this analysis, these findings should be interpreted cautiously and require validation in independent cohorts before translational relevance can be established.

#### 4.2.5. AMF Analysis Within High-Power Fields

The high-power field (HPF) is a standard unit in pathology practice. We therefore performed HPF-related analyses and found that AMFs are more strongly associated with HPF-based metrics. We defined an HPF as a fixed $3\;{\mathrm{mm}}^{2}$ window containing the maximum number of mitotic figures within the tumor area, identified using a dense sliding-window strategy with 95% area overlap. We then counted the number of AMFs within these HPF regions and performed correlation and survival analyses for the same early-stage (I–II) TCGA-BRCA cases.

Because this HPF definition intentionally selects the window with the maximum number of mitotic figures, it is biased toward highly proliferative hotspot regions by design. Therefore, the following AMF correlations should be interpreted as hotspot-associated patterns rather than evidence of preferential AMF spatial enrichment.

**Figure 6:**
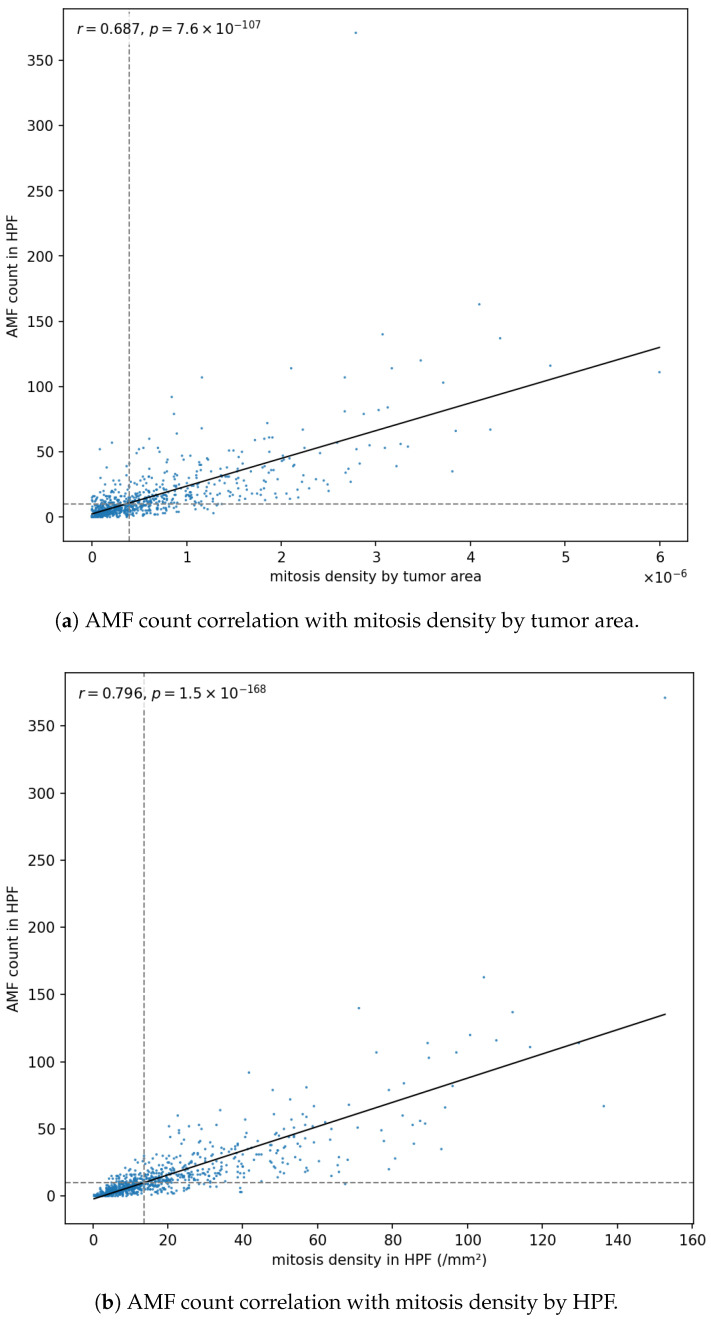
Correlation analysis of AMF count with mitosis density by tumor area and HPF for Stage I–II TCGA-BRCA cases. (**a**) AMF count correlation with mitosis density by tumor area. (**b**) AMF count correlation with mitosis density by HPF.

Figure 6a shows the correlation between AMF count and whole-tumor mitosis density (r=0.687), indicating a moderate association between AMF burden and overall proliferative activity. Figure 6b shows that AMF count within HPF regions demonstrates a stronger correlation with HPF-based mitosis density (r=0.796). This elevated correlation indicates that AMF burden co-varies with locally proliferative hotspot activity as captured by the HPF definition. Moreover, the moderate correlation in Figure 6a with whole-tumor mitosis density suggests that HPF serves as a proxy for whole tumor area, though this relationship could potentially be strengthened through more advanced HPF selection strategies.

**Figure 7:**
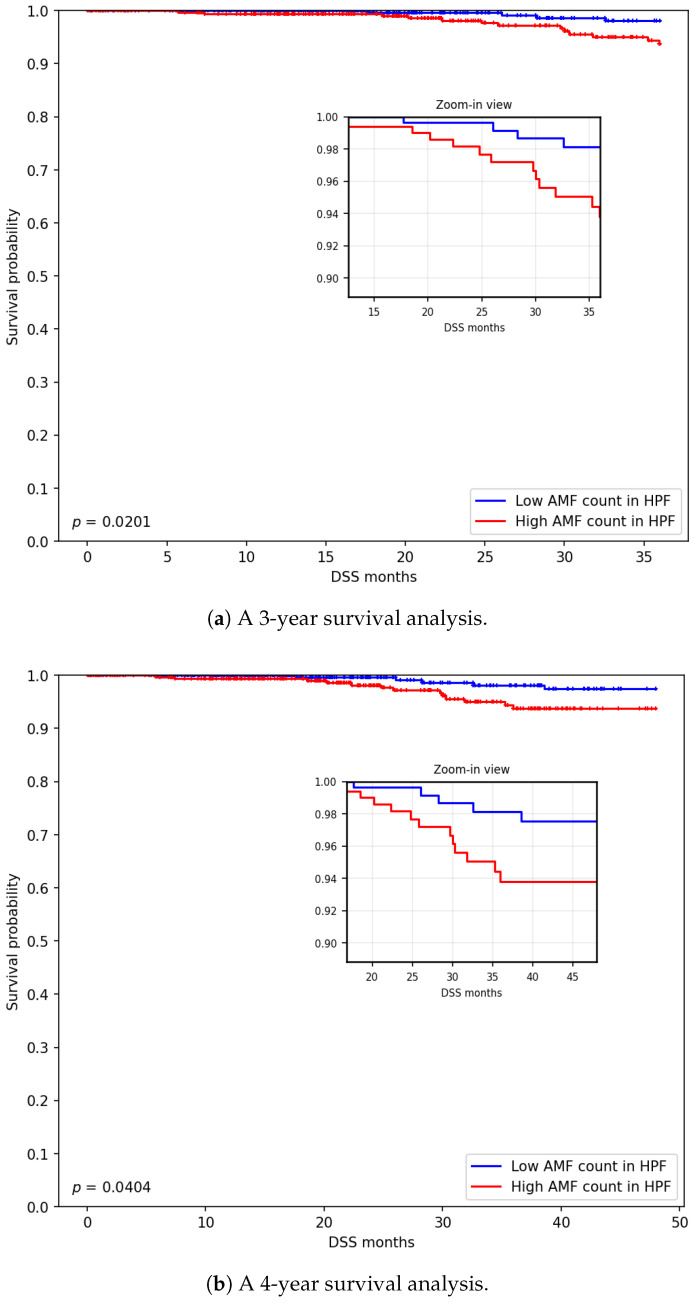
Survival analysis performed on the TCGA-BRCA early-stage (I and II) samples, stratified by AMF count within HPF regions (3 mm^2^ window). (**a**) The 3-year and (**b**) 4-year disease-specific survival (DSS) curves, with groups split by AMF count within HPF regions. Patients with high AMF count within HPF regions (red) show lower DSS compared to low AMF count within HPF regions (blue).

We further evaluated prognostic relevance using the AMF count within HPF regions. As shown in Figure 7a, early-stage patients with lower AMF counts within HPF regions exhibit significantly improved 3-year disease-specific survival (*p* = 0.0201). Figure 7b shows that this prognostic pattern persists over 4 years (*p* = 0.0404), consistent with the whole-tumor analysis. This exploratory result suggests that AMF burden within HPF-defined hotspot regions may carry clinically relevant prognostic information and may provide complementary risk-stratification value alongside whole-tumor mitosis density.

#### 4.2.6. Computation Efficiency

Computational efficiency is essential for pipelines intended for future clinical workflow evaluation or large-scale research applications. Hence, we further evaluated the computational efficiency of our pipeline on all TCGA-BRCA slides using the following configuration: (1) a YOLOv11 large variant as the first-stage detector; (2) an EfficientNet-b0 model as the second-stage classifier; and (3) an EfficientViT-L2 model as the third-stage AMF/NMF classifier.

Across all TCGA-BRCA slides processed on a single RTX 4090 GPU, the end-to-end per-slide processing time showed a median of 104.91 s and a mean of 107.18 s. The total processing time for the full cohort was 113,821.07 s, corresponding to 31.62 h. In terms of practical throughput, 59.7% of slides were processed within 2 min, 89.7% within 3 min, and 96.9% within 4 min. Notably, with further engineering efforts and optimizations such as multi-GPU parallel processing and higher GPU utilization, the processing time can be further reduced. Additionally, a mid-range GPU with only 8 GB Video RAM (VRAM) is also capable of running our pipeline with the same configuration.

## 5. Discussion

In this work, we present a highly efficient and scalable three-stage pipeline for fast mitosis candidate detection, mitosis versus non-mitosis classification, and AMF/NMF classification. We validate our approach on three benchmarks: (1) mitosis detection on MIDOG++, (2) AMF/NMF classification on MIDOG 2025 Track 2, and (3) translational relevance on the TCGA-BRCA cohort via survival analyses. Our results demonstrate strong technical performance and provide proof-of-concept evidence that mitosis-derived features may have translational relevance in early-stage TCGA-BRCA.

In early-stage (I–II) TCGA-BRCA cases, mitosis density in the tumor area estimated by our pipeline was associated with survival outcomes in Kaplan–Meier analysis and showed modest prognostic discrimination in Cox modeling. These results support the potential translational relevance of automated mitosis quantification as a slide-level biomarker aligned with standard pathology concepts of tumor proliferation.

Since high-power field (HPF) or hotspot selection is routine in pathology practice, we further investigated the translational relevance of our AMF classification by examining AMF burden within a fixed 3 mm^2^ window of maximum mitotic density, using a dense sliding-window approach with 95% overlap. We found that AMF counts in HPF regions showed a stronger correlation with HPF-based mitosis density (r=0.796) than with whole-tumor mitosis density (r=0.687), suggesting that AMF burden co-varies with highly proliferative hotspot regions. It is important to note that because HPFs here were defined as maximum-mitosis windows by design, our observations reflect hotspot-associated patterns rather than evidence of true spatial enrichment of AMFs beyond what would be expected in high-proliferation regions. Nevertheless, when stratifying patients by HPF-AMF counts in survival analysis, we observed a similar prognostic difference between the high and low groups, indicating that AMF quantification may provide prognostic information alongside mitosis density.

A major focus of this work is the computational efficiency of our pipeline, which makes automated mitosis analysis more practical for large-scale translational pathology studies. In a large-scale evaluation on the TCGA-BRCA dataset, containing roughly one thousand slides, our pipeline processed each slide in only a few minutes and remained deployable on a mid-range GPU with 8 GB of video memory, making it both time- and cost-efficient for many institutions.

This study has several limitations. First, because this is an initial study introducing a highly efficient mitosis analysis pipeline, the TCGA survival analysis should be interpreted as a proof-of-concept translational evaluation rather than definitive clinical validation. Although the Cox models showed consistent improvements in discrimination, the number of disease-specific events in the early-stage fixed-horizon setting was limited, which reduces statistical power and may attenuate statistical significance. Second, the HPF selection analysis remains a proof-of-concept demonstration, and more advanced HPF selection strategies will be studied in future work. Third, mitotic morphology is only partially exploited in the current framework; beyond detection and AMF/NMF categorization, richer morphologic characterization may provide additional clinical and biological insight. Therefore, these findings should not be interpreted as evidence of clinical readiness, and validation in independent cohorts with larger event counts is needed.

The proposed pipeline is intended as a computational decision-support and large-scale research tool, not as an autonomous clinical diagnostic system. In clinical use, Stage 2 should be interpreted as a prioritization or false-positive suppression step rather than an irreversible discard decision. AI-retained, borderline, and AI-suppressed candidates should remain available for expert pathologist review, and prospective pathologist-in-the-loop validation is required before future clinical workflow use.

For future work, we will extend our pipeline to include more tumor types and additional real-world clinical cohorts through ongoing collaborations to further validate its generalizability. We also believe that mitosis morphology analysis provides deeper insight than mitosis detection and counting alone, since mitotic morphology is linked to underlying genetic features. For example, AMFs include subtypes such as bridging and triangular forms, which may be associated with genomic alterations, chromatin instability, or DNA damage. Hence, we will explore opportunities to extend our pipeline to become more morphology-aware. Furthermore, our early exploration of high-power field (HPF) selection revealed that careful HPF selection is critical for accurate diagnosis and prognosis analysis. Therefore, we will further investigate more advanced HPF selection strategies and their impact on diagnostic and prognostic outcomes.

## 6. Conclusions

This study presents an efficient three-stage pipeline for whole-slide mitosis analysis, integrating mitosis candidate detection, mitosis verification, and atypical versus normal mitotic figure classification. Across benchmark evaluations, the pipeline achieved competitive mitosis detection performance while maintaining substantially lower computational cost than heavier detector- or foundation-model-based alternatives. The Stage 3 classifier further enabled AMF/NMF analysis, extending the pipeline beyond mitosis counting toward richer characterization of mitotic morphology.

Applied to TCGA-BRCA, the pipeline processed whole-slide images at practical throughput and provided proof-of-concept evidence that mitosis-derived features may offer modest incremental prognostic information in early-stage breast cancer. These survival findings should be interpreted as exploratory rather than definitive clinical validation, and future studies in independent cohorts with larger event counts are needed. Overall, the proposed framework offers a scalable and computationally accessible foundation for large-scale mitosis analysis and future translational pathology studies.

## Figures and Tables

**Table 1:** Comparison of representative object detection models.

Model	Variants ^1^	Params (M)	GFLOPs ^2^	COCO mAP
**YOLOv11**	n/s/m/l	2.6/9.4/20.1/25.3	6.5/21.5/68.0/86.9	39.5/47.0/51.5/53.4
**RT-DETR v2**	s/m/l/x	20/36/42/76	60/92/136/259	48.1/49.9/53.4/54.3
**Faster R-CNN**	R50-FPN	41.8	269.1	37.0

^1^ Model variants by scale: n: nano; s: small; m: medium; l: large; x: extra large. ^2^ All with 640 × 640 input image size. Faster R-CNN GFLOPs is estimated by torch.profiler in PyTorch framework.

**Table 2:** Distribution of tumor types and slides of combined dataset.

Tumor Type	Number of Slides
* Cutaneous mast cell tumor	50
* Lung carcinoma	44
* Lymphosarcoma	55
* Soft tissue sarcoma	100
** Breast carcinoma	150
** Melanoma	49
** Neuroendocrine tumor	55
**Total**	**503**

* Canine, ** Human.

**Table 3:** AMF and NMF samples across cancer types for combined training set.

Cancer Type	Sample Num	AMF	NMF	AMF %
* Cutaneous mast cell tumor	2327	351	1976	15.10%
* Lung carcinoma	855	110	745	12.90%
* Lymphoma	3959	317	3642	8.00%
* Soft tissue sarcoma	1286	210	1076	16.30%
** Breast carcinoma	3722	832	2890	22.40%
** Melanoma	1150	271	879	23.60%
** Neuroendocrine tumor	639	85	554	13.30%
**Total**	**13,938**	**2176**	**11,762**	**15.60%**

* Canine, ** Human.

**Table 4:** Overall MIDOG++ test set performance with YOLOv11 variants as first stage model.

Stage 1 Models	No. of Params	GFLOPs	Stage 1 Only			With Stage 2 Classifier		
	(M)		P ^1^	R ^1^	F1	P	R	F1
YOLOv11-l	25.3	86.9	0.41	0.91	0.57	**0.71**	**0.82**	**0.76**
YOLOv11-m	20.1	68.0	0.38	**0.92**	0.54	0.67	**0.82**	0.74
YOLOv11-s	9.4	21.5	0.40	0.89	0.55	0.69	0.80	0.74
YOLOv11-n	2.6	6.5	0.42	0.86	0.57	0.69	0.78	0.73

^1^ P: Precision; R: Recall. Bold values indicate the best-performing value in the corresponding column or tied best result.

**Table 5:** Stage 2 mitosis/non-mitosis classification performance comparison on MIDOG++ test set.

Model	Accuracy (%)	F1	No. of Params (M)	GFLOPs ^1^
EfficientNet-b0	84.69	**0.85**	5.3	0.25
EfficientNet-b1	84.67	**0.85**	7.8	0.38
EfficientNet-b2	**84.88**	**0.85**	9.2	0.43
EfficientNet-b3	84.43	0.84	12.0	0.63
ResNet-18	82.80	0.83	11.7	1.19

^1^ GFLOPs were estimated using 128×128 input patches. Bold values indicate the best-performing value in the corresponding column or tied best result.

**Table 6:** Domain-wise F1 comparison on the MIDOG++ benchmark for different mitosis detection approaches.

Cancer Type	RetinaNet [8]	Faster R-CNN [46]	OMG-Net ^†^ [24]	YOLOv11-L + EfficientNet-B0 (Ours)
* Cutaneous mast cell tumor	0.82	0.85	**0.86**	**0.86**
* Lung carcinoma	0.68	0.65	**0.69**	**0.69**
* Lymphoma	0.73	**0.78**	0.76	**0.78**
* Soft tissue sarcoma	0.69	0.73	**0.74**	0.68
** Breast carcinoma	0.71	0.74	**0.85**	0.74
** Melanoma	0.81	0.80	**0.83**	0.78
** Neuroendocrine tumor	0.59	0.58	**0.64**	0.55
**Domain-wise Mean**	0.72	0.73	**0.77**	0.73

* Canine cancer type; ** Human cancer type. ^†^ **Note:** OMG-Net was trained using a larger multi-source dataset and uses SAM-based cell proposal generation with additional mask curation. Bold values indicate the best-performing value in each row or tied best result.

**Table 7:** Computational overhead comparison of representative detection approaches.

Method (First-Stage Model)	Stage 1 Detector GFLOPs	Relative Cost
Ours (YOLOv11-N)	6.5	0.07×
Ours (YOLOv11-S)	21.5	0.25×
Ours (YOLOv11-M)	68.0	0.78×
Ours (YOLOv11-L)	86.9	1.00×
RetinaNet (R50-FPN)	151.54	1.74×
Faster R-CNN (R50-FPN)	269.1	3.10×
OMG-Net (SAM ViT-H encoder) ^†^	∼1164.8	13.40×

^†^ SAM ViT-H encoder cost is reported as 2982 GFLOPs at 1024 × 1024 input and estimated at 640 × 640 by area normalization: $2982\times (640^{2}/1024^{2})\approx 1164.8$ GFLOPs. This estimate excludes prompt encoding, mask decoding, dense mask post-processing, and downstream ResNet18 classification, so the full OMG-Net cost is expected to be higher.

**Table 8:** Performance of Stage 3 AMF/NMF classifier on MIDOG 2025 (Track 2) final test set.

	Across 12 Domains		Across
	Mean	Std.	All Samples
**ROC-AUC**	0.953	0.019	0.962
**Accuracy**	0.905	0.031	0.909
**Sensitivity**	0.846	0.131	0.879
**Specificity**	0.899	0.049	0.915
**BA**	0.873	0.054	0.897

**Table 9:** Exploratory comparison of nested Cox models for stage I–II TCGA-BRCA by follow-up duration.

Follow-Up (Months)	Model	C-Index (95% CI)	ΔC-Index vs. Baseline	*p*-Value
36	1. Clinical (baseline)	0.769 (0.652–0.870)	-	-
	2. Clinical + Nuclei	0.781 (0.671–0.871)	0.012	0.5352
	3. Clinical + Nuclei + Mitosis	**0.802** (0.702–0.883)	0.033	0.1052
48	1. Clinical (baseline)	0.777 (0.641–0.865)	-	-
	2. Clinical + Nuclei	0.787 (0.667–0.867)	0.010	0.5576
	3. Clinical + Nuclei + Mitosis	**0.802** (0.695–0.881)	0.025	0.0852

Note: C-index 95% confidence intervals were estimated using patient-level bootstrap resampling. ΔC-index values were calculated relative to the clinical baseline model at the same follow-up duration. The *p*-values correspond to nested likelihood-ratio tests: Model 2 vs. Model 1 and Model 3 vs. Model 2. Bold values indicate the best-performing C-index at the same follow-up duration.

## Data Availability

The code for the web-based mitosis analysis pipeline described in this study will be released on GitHub link: https://github.com/XuanQi-NCI/mitosis_analysis_web_pipeline (accessed on 26 May 2026). Public datasets used in this work are cited in the manuscript.
